# Low birth weight influences the postnatal abundance and characteristics of satellite cell subpopulations in pigs

**DOI:** 10.1038/s41598-020-62779-1

**Published:** 2020-04-09

**Authors:** K. Stange, C. Miersch, G. Sponder, M. Röntgen

**Affiliations:** 1 Leibniz Institute for Farm Animal Biology, Institute of Muscle Biology and Growth, 18196 Dummerstorf, Germany; 2 Freie Universität Berlin, Institute of Veterinary Physiology, 14163 Berlin, Germany

**Keywords:** Intrauterine growth, Metabolism, Muscle stem cells

## Abstract

Low birth weight (LBW) can cause lifelong impairments in muscle development and growth. Satellite cells (SC) and their progeny are crucial contributors to myogenic processes. This study provides new data on LBW in piglets combining insights on energy metabolism, muscle capillarization and differences in SC presence and function. To this aim, muscle tissues as well as isolated myogenic cells of 4-day-old German Landrace piglets were analyzed. For the first time two heterogeneous SC subpopulations, which contribute differently to muscle development, were isolated from LBW pigs by Percoll density gradient centrifugation. The muscles of LBW piglets showed a reduced DNA, RNA, and protein content as well as lower activity of the muscle specific enzymes CK, ICDH, and LDH compared to their normal birth weight siblings. We assume that deficits in energy metabolism and capillarization are associated with reduced bioavailability of SC, possibly leading to early exhaustion of the SC reserve cell pool and the cells’ premature differentiation.

## Introduction

The pig remains one of the most important farm animals worldwide and nowadays also represents a highly appreciated model system for scientific studies. In the past decades pigs were mainly selected for economic traits like reproductive fitness, growth performance, or litter size. An analysis of wild boar litters in Europe showed a mean litter size between 4.75–6.28 piglets^1^. In contrast, in domestic pigs a mean litter size of 10.9 piglets in 1992 was further increased by 12% to 12.2 piglets already in 2001^2^. Varona and colleagues even reported a mean litter size of 14.23 piglets in Landrace pigs^3^. In polytocous species uterus capacity is a limiting factor, becoming important from day 25 of gestation on^4,5^, and more than 14 embryos can be considered as intrauterine crowding^5^ possibly leading to intrauterine competition and retardation of prenatal growth^5,6^.

Bigger litter sizes can lead to intrauterine growth retardation (IUGR) due to insufficient development of the placenta in relation to the number of embryos, which are not sufficiently supplied with oxygen and nutrients^5^. Consequently, birth weight variation (and with this also weaning weight variability) increases in large litters; and with increasing litter birth weight more low birth weight (LBW) piglets are born^2,7,8^. Several important factors like birth weight, weaning weight, and gender contribute to postnatal growth performance of pigs^9^, whereas the birth weight is the earliest and most easily accessible one. Up to 15–20% of pigs exhibit a low birth weight^10^ associated with developmental disadvantages compared to their normal birth weight (NBW) litter mates. Low birth weight, for instance caused by IUGR, is also relevant for other species like sheep or cattle^10,11^ and for humans, where for example in the U.S. approximately 5–15% of all children have to cope with IUGR^11–13^.

Some LBW pigs show compensatory growth but exhibit a higher risk for infection diseases and in general their survival rate is still reduced^9,14,15^. Memory deficits in LBW piglets as well as their reduced willingness to play could be an indicator for a decreased animal welfare^14,16,17^. Moreover, a decrease in birth weight correlates with a poor postnatal growth rate characterized by a lower feed conversion ratio and reduced daily weight gain as well as with a lower carcass and meat quality after slaughter^2,15^. Analyses of whole body composition revealed a lower fat and protein but higher water content^15^. LBW pigs weighed 12% less at weaning and needed 12 days longer to reach slaughter weight^2^.

As skeletal muscle development is crucial for pig husbandry, the manifestation of a musculoskeletal phenotype in pigs is of special interest. Skeletal muscle is mainly composed of multinucleated myofibers which are able to contract. By multiple fusion of myogenic cells, primary fibers are formed during embryonic development between day 35 and day 60 of gestation and secondary fibers are formed during fetal development (day 55–90 of gestation)^18,19^. These two prenatal waves of fiber development mainly determine muscle fiber number which is mostly fixed at birth^20^. In a third phase of fiber development so called tertiary fibers are perinatally formed in the pig although their quantitative contribution to muscle development is thought to be rather low^21,22^. Muscle growth after birth is mainly achieved by hypertrophic growth of already existing muscle fibers. In piglets with LBW the percentage of muscle tissue in whole body weight is reduced^15^. The musculoskeletal phenotype developed by a large number of LBW piglets can lead to lifelong impairment in muscle growth^15,23^ typically associated with the formation of less muscle fibers with a larger diameter. Less myogenic cell nuclei were found within the muscle fibers of LBW piglets compared to NBW piglets^2,15,24^.

Postnatally, satellite cells (SCs) and their progeny play a crucial role in muscle development, growth, and regeneration. SCs provide cell nuclei for muscle fiber fusion and growth and are able to terminally differentiate as well as to self-renew^25,26^. Adult SCs are mostly quiescent and were originally termed according to their localization between the sarcolemma and the basal lamina^27^. Quiescent SCs express the transcription factor Pax7; and also Myf5 when becoming committed to the myogenic lineage^26,28–31^. Activated SCs undergo proliferation and later on myogenic differentiation to finally form new myofibers or fuse to existing myofibers^21,25,32^. Proliferating myoblasts, in addition to Pax7 and Myf5, start to express MyoD. Myocytes downregulate Pax7 and upregulate the differentiation marker Myogenin (MyoG) (reviewed in^26^). In adult pigs only 2–5% of cell nuclei are sublaminal myonuclei^30,33^ which was shown to differ dramatically in early postnatal development. During the first week of life up to 60% of cells isolated from piglet muscle belong to the SC population whereas 90% of them are in a proliferating state^25^. Between weeks 7 and 21 of life the percentage of SCs lowers to 30–40% but still more than 70% of these cells are not quiescent yet. Only about one third of the cells is able to undergo myogenic differentiation leading to the hypothesis that the remaining SC population forms and maintains the SC stem cell pool^30,34,35^. The size of the SC pool established in early development is crucial for lifelong muscle performance^30^.

For a more comprehensive understanding of long term effects caused by birth weight variations, tissue and muscle cell analyses should be interrelated. This study provides new and updated data on LBW pigs and also aims to connect muscle tissue properties to characteristics of two distinct myogenic cell subpopulations which have never been isolated from LBW piglets before.

## Results

### General data on litters of German landrace pigs

We first aimed to give an overview over the current litter characteristics of German Landrace pigs. 64 litters consisting of 1057 pigs were analyzed regarding birth weight, litter size and gender (Table 1). Including dead or sacrificed piglets, in average 16.53 piglets were born per litter. According to this value litters were categorized into two groups and large litters were defined as consisting of more than 16 piglets. 57.8% of all litters allotted to that category. Both litter groups were compared statistically. The mean birth weight amounted to 1.32 kg over all litters and was significantly decreased in large litters (1.28 kg) compared to smaller litters (1.39 kg). Gender distribution did not depend on litter size.

**Table 1:** Characteristics of litters and newborn piglets of German Landrace piglets.

		All litters (8–21 pigs)	Litters ≤16 pigs		Litters >16 pigs
Pigs/litter		16.53 ± 2.63	14.04 ± 1.72	P ≤ 0.001	18.35 ± 1.38
Birth weight		1.32 kg ± 0.16	1.39 kg ± 0.17	P = 0.003	1.28 kg ± 0.14
% male pigs		48.67 ± 12.04	46.20 ± 14.84	P = 0.216	50.48 ± 9.32
% female pigs		51.33 ± 12.04	53.80 ± 14.84	P = 0.216	49.52 ± 9.32
Birth weight categories	Runt (<0.8 kg)	6.53% 0.64 kg ± 0.09	4.79% 0.62 kg ± 0.10	P = 0.062 P = 0.211	7.80% 0.64 kg ± 0.08
	Low (0.8–1.15 kg)	24.11% 1.01 kg ± 0.05	20.13% 1.02 kg ± 0.05	P = 0.024 P = 0.058	27.02% 1.00 kg ± 0.05
	Middle (1.16–1.55 kg)	44.67% 1.37 kg ± 0.06	43.27% 1.39 kg ± 0.06	P = 0.020 P = 0.020	45.70% 1.35 kg ± 0.06
	High (>1.55 kg)	24.68% 1.72 kg ± 0.08	31.81% 1.72 kg ± 0.10	P = 0.066 P = 0.310	19.48% 1.71 kg ± 0.07

64 litters with a total of 1057 pigs of German Landrace were evaluated and divided into two groups according to average number of piglets/litter. Litters with more than 16 piglets showed a significantly lower mean birth weight. The percentage of male and female piglets did not depend on litter size.

Runts were defined as piglets with a birth weight <800 g (72 piglets); the remaining animals were categorized into three birth weight groups: 25% low (260 piglets), 50% middle (476 piglets) and 25% high (249 piglets) according to quartiles of frequency distribution. For statistical analysis data from small and large litters were compared, respectively. In order to analyse birth weight distribution (%), one tailed t-test (low, middle) or Mann-Whitney Rank Sum test (runt, high) was performed. For statistical analysis of birth weight (kg) one tailed t-test was performed.

To further analyze birth weight distribution, piglets were categorized into runts (birth weight below 800 g) and three groups according to quartiles of frequency distribution (low, middle, and high). In analyzed litters, this categorization revealed a birth weight of 1.16–1.55 kg for the middle birth weight group. Distribution of piglets with low, middle, and high birth weight within birth weight categories strongly depended on litter size. The birth weight significantly decreased for middle birth weight piglets between small and large litters. Low birth weight piglets tended to be slightly lighter in large litters. In large litters significantly more piglets with low birth weight were born. Accordingly, fewer piglets with high birth weight were observed in large litters.

### (Muscle) weight of low birth weight piglets used for the study

**Figure 1:**
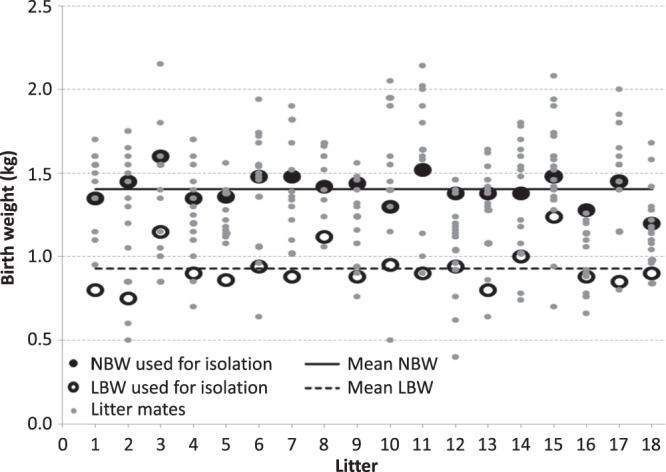
Birth weight distribution in NBW and LBW piglets. Birth weight distribution in litters from which NBW (n = 18) and LBW (n = 18) piglets were chosen. The mean birth weight of animals (all female) used for further analyses (e.g. cell isolation) is depicted as a solid (NBW) or dashed (LBW) line. Average litter size was 16.33 ± 2.95 piglets per litter.

German Landrace piglets used for studying muscle development were selected according to birth weight in order to compare low and normal birth weight piglets at day 4 of age. Female piglets, which were vital, phenotypically healthy, and gained weight within this time frame, were included. Thereby the lightest pig was selected as LBW. The corresponding NBW pig was chosen from the same litter and its birth weight should be near the mean birth weight of this litter (Fig. 1). The birth weight as well as the body weight at day 4 (time point of sacrifice) differed significantly between NBW and LBW piglets used in this study (Table 2). Piglets with normal birth weight were able to gain more body weight. Interestingly, linear regression analysis at day 4 of age revealed a considerable correlation between the size of LD muscle and body weight (R^2^ = 0.77, data not shown). In contrast, the size of the SM muscle did not strongly correlate with body weight (R^2^ = 0.52, data not shown). SM and LD muscles in NBW piglets have a higher mass and LD muscle also accounts for a greater percentage of total body weight (Table 2).

**Table 2:** Body and muscle weight are decreased in LBW piglets.

	NBW		LBW
Birth weight	1.40 kg ± 0.08	P = 9.5*10^−12^	0.92 kg ± 0.12
Weight at isolation (day 4)	1.91 kg ± 0.22	P = 1.2*10^−9^	1.19 kg ± 0.15
Increase in weight (between birth and day 4)	36.81% ± 12.78	P = 0.073	29.92% ± 17.94
Muscle weight SM	13.68 g ± 4.19	P = 6.3*10^−6^	9.14 g ± 3.43
% muscle weight SM/body weight (at day 4)	0.71% ± 0.19	P = 0.0675	0.76% ± 0.25
Muscle weight LD	28.54 g ± 8.00	P = 9.8*10^−8^	15.79 g ± 4.80
% muscle weight LD/body weight (at day 4)	1.48% ± 0.32	P = 0.013	1.32% ± 0.03

Body and muscle weight of female piglets used for the study (NBW: n = 18, LBW: n = 18). LBW piglets showed significantly lower body weight at birth and at cell isolation (day 4 of life) and also reduced percentage weight gain during this time. The mass of SM and LD muscles was reduced in LBW piglets. For statistical analysis one tailed paired t-test was performed.

### Serum metabolite concentrations in normal and low birth weight piglets

Serum analysis can provide information on the nutrient status of an animal. Therefore, immediately after slaughter serum samples were taken and analyzed for non-esterified fatty acids (NEFA), triglycerides and glucose (Table 3). Circulating NEFA and triglyceride levels were significantly elevated in LBW compared with NBW piglets. Glucose levels did not differ between piglets with different birth weights.

**Table 3:** Serum analysis of 4-day-old German Landrace piglets with low or normal birth weight.

	NBW		LBW
NEFA (µmol/l)	340 ± 97	P = 0.006	454 ± 174
Triglyceride (mmol/l)	1.53 kg ± 0.56	P = 0.008	2.17 kg ± 1.27
Glucose (mmol/l)	6.85 ± 1.47	P = 0.258	6.50 ± 1.58

Serum was collected from animals with normal (n = 19) or low (n = 19) birth weight at day 4 of life. Levels of non-esterified fatty acids (NEFA) and triglycerides were significantly elevated in LBW piglets. Glucose levels did not differ between NBW and LBW piglets. For statistical analysis one tailed paired t-test (NEFA, triglyceride) or Wilcoxon Signed Rank Test (Glucose) was performed.

### Alterations in muscle tissue properties of low birth weight piglets

**Figure 2:**
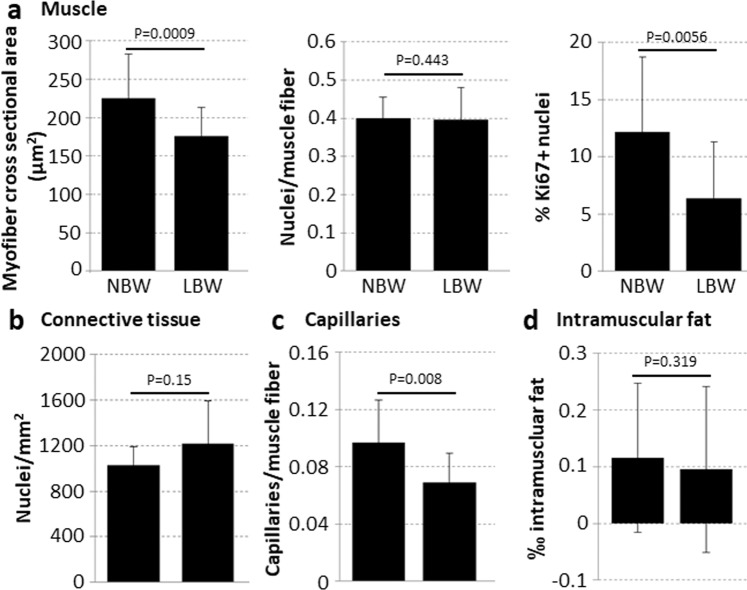
Histomorphological characteristics of muscle samples from 4-day-old piglets. Cryosections of SM muscle (NBW: n = 8, LBW: n = 8) were stained to visualize muscle fibers and connective tissue (eosin), capillaries (alkaline phosphatase) and intramuscular fat (Oil Red). For staining of proliferating muscle nuclei (Ki67+) LD muscle of 4-day-old piglets (NBW: n = 12, LBW: n = 12) was used. For statistical analysis Wilcoxon Signed Rank test (capillaries) or onetailed paired t-test was performed. (**a**) Myofibers of LBW piglets showed a reduced cross sectional area compared to those of NBW piglets, and counted nuclei were less proliferating as seen by staining of Ki67. (**b**) Number of cell nuclei in connective tissue did not differ between NBW and LBW piglets. (**c**) In LBW piglets significantly fewer capillaries per muscle fiber were found. (**d**) The proportion of intramuscular fat in evaluated sections was very low and did not differ between NBW and LBW piglets.

Histomorphological characteristics were analyzed to obtain information on muscle fiber development, capillarization, and proportion of connective and fat tissue (Fig. 2). In addition, the number of proliferating muscle cells was determined.

Some parameters were extensively studied in piglets with different birth weight and therefore also serve as a quality control for animal selection. Myofiber cross sectional area (FCSA) was smaller in samples from LBW than in those from NBW piglets (Fig. 2a). Interestingly, significantly less cell nuclei within muscle tissue expressed the proliferation marker Ki67 in piglets with LBW. The number of nuclei per myofiber (Fig. 2a) and nuclei in connective tissue (Fig. 2b) did not differ between birth weight groups. However, significantly fewer capillaries per muscle fiber were found in LBW piglets (Fig. 2c). The percentage of intramuscular fat is very low in neonatal piglets and did not differ depending on birth weight (Fig. 2d). Analysis of tissue composition also showed no significant difference in fat content of LD muscle at day 4 of age (NBW 3.13 ± 0.87%, LBW 3.54 ± 1.31%, n ≥ 5). Dry matter and water content did not differ between animals as well.

**Figure 3:**
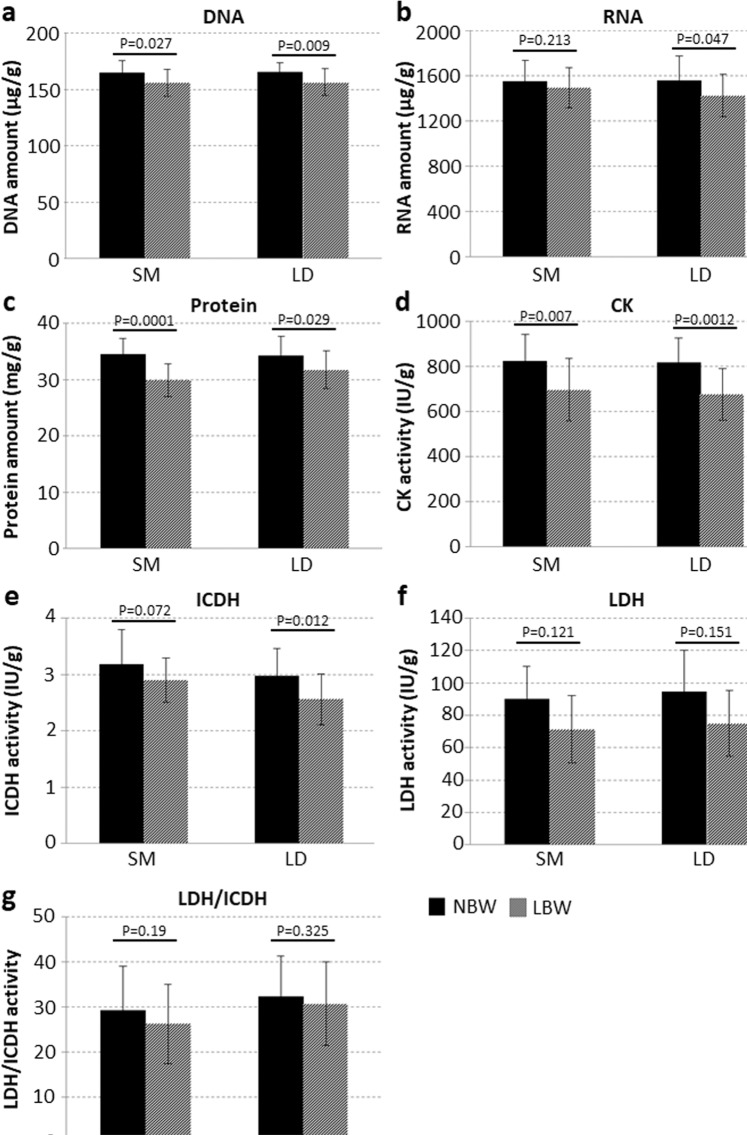
Biochemical analyses of muscle tissue. Tissue samples from SM and LD muscles of 4-day-old female piglets with normal or low birth weight were analyzed (NBW: n = 14, LBW: n = 14). For statistical analysis Mann-Whitney Rank Sum test (DNA LD) or one tailed t-test was performed. The amount of DNA (**a**), RNA (**b**), and total protein (**c**) as well as the activity of creatine kinase (CK, **d**) and isocitrate dehydrogenase (ICDH, **e**) were significantly reduced in SM and/or LD muscle of LBW piglets compared to NBW piglets. Lactate dehydrogenase (LDH) activity (**f**) and the ratio of LDH/ICDH activity (**g**) did not differ between NBW and LBW piglets.

Next, muscle tissue samples from LBW and NBW piglets were analyzed regarding nucleic acid and protein content as well as the activity of muscle specific enzymes. DNA and RNA amount was significantly lowered in SM and/or LD muscle in case of a LBW (Fig. 3a,b). Protein content was also decreased in LBW compared with NBW piglets (Fig. 3c). Considerably more creatine kinase (CK, Fig. 3d) and isocitrate dehydrogenase (ICDH, Fig. 3e) were found in NBW compared with LBW piglets. In contrast, lactate dehydrogenase activity (LDH, Fig. 3f) and LDH/ICDH ratio (Fig. 3g) were similar in both groups.

### Myogenic characteristics of freshly isolated SC in low birth weight piglets

**Figure 4:**
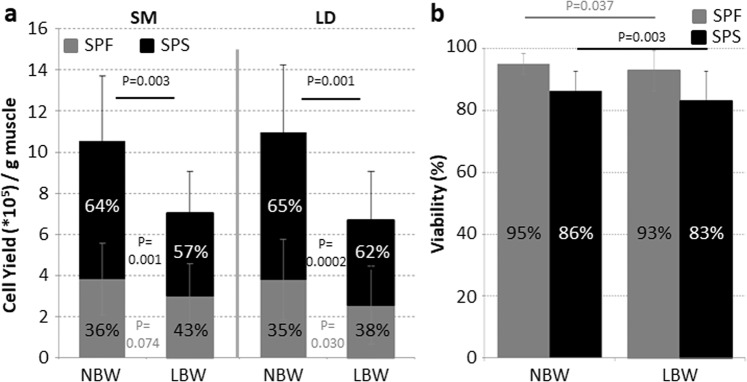
Cell yield and viability after isolation. SC subpopulations were isolated from SM and LD muscle using Percoll gradient centrifugation. (**a**) Total cell yield (NBW: n = 17, LBW: n = 17) was significantly lower in muscle from LBW piglets compared to NBW piglets. Less cells of the subpopulation fast (SPF) and subpopulation slow (SPS) were isolated from LBW piglets. SPS cells account for a smaller percentage in LBW than in NBW piglets. For statistical analysis one tailed paired t-test was performed. (**b**) A high viability was reached for all isolated cells, although it was significantly lower in LBW piglets for both subpopulations (NBW: n = 18, LBW, n = 18). For statistical analysis Wilcoxon Signed Rank test (SPF) or one tailed paired t-test (SPS) was performed.

SC/MPC were isolated from SM and LD muscle of 4-day-old piglets with normal or low birth weight and separated into subpopulation fast (SPF) and subpopulation slow (SPS), named after their proliferative potential^36^. The overall cell yield per gram muscle from LBW piglets was significantly reduced by 33% in SM and 39% in LD muscle (Fig. 4a) and the cell yield in both subpopulations was decreased in these animals as well. As described in our previous study for NBW piglets^36^, also in LBW piglets the majority of cells was found in the SPS. Viability of all isolated cells clearly exceeds 80%, although cells of both subpopulations showed a significantly reduced viability when isolated from LBW piglets (Fig. 4b).

**Figure 5:**
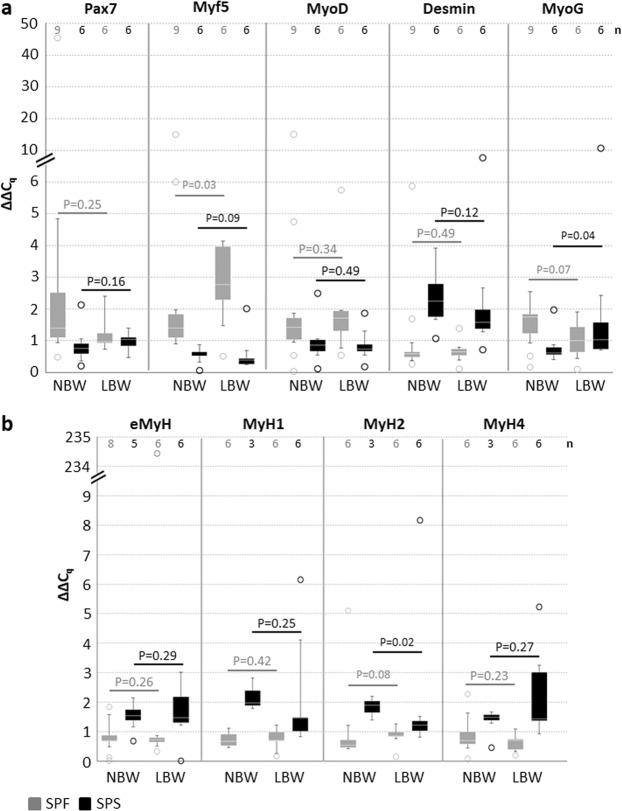
Expression of myogenic genes. Gene expression was analyzed via qRT-PCR in freshly isolated cells. Typical myogenic genes were expressed in both isolated subpopulations of NBW and LBW piglets. ΔΔC_q_ values are shown as Box-Whisker plots with the maximum 1.5 of the interquartile range (Q_1_-Q_3_), outliers are presented as circles. For statistical analysis Mann-Whitney Rank Sum test (Pax7 SPF, Myf5 SPF, MyH1 SPS) or one tailed t-test was performed in order to compare expression levels within subpopulation between NBW and LBW piglets. Number of animals included is given in the respective upper part (n). (**a**) *Myf5* expression was increased in SPF cells of LBW piglets, but decreased in SPS cells. In contrast, *MyoG* expression was lowered in SPF cells and enhanced in SPS cells of LBW piglets. Expression of *Pax7*, *MyoD1* and *Desmin* remained unchanged between NBW and LBW piglets. (**b**) Expression of MyH2 was significantly lower in SPS cells of LBW piglets. *MyH1*, *MyH4* and *eMyH* expression was comparable in NBW and LBW piglets.

To ascertain the status-quo of freshly isolated cells from 4-day-old NBW and LBW piglets, the expression of important myogenic genes was determined in a semi-quantitative manner. *Pax7*, *Myf5*, *MyoD*, *Desmin* and *MyoG* were used as markers for specific steps of cell development and commitment in myogenesis (Fig. 5a). Furthermore, members of the Myosin gene family were analyzed (Fig. 5b). The expression of the transcription factor *Myf5* was significantly enhanced in SPF cells of LBW piglets, whereas expression tended to be decreased in corresponding SPS cells. In contrast, *MyoG*, a marker for early differentiating cells, was slightly reduced in SPF cells and significantly enhanced in SPS cells of LBW piglets. Of the analyzed Myosin genes, only *MyH2* showed a significant difference referred to birth weight – its expression was lowered in SPS cells of LBW piglets.

## Discussion

In this study we provide data on litters of 64 German Landrace sows from our pig facility. In the evaluated population, the average number of pigs per litter (16.53) was higher than reported by the *Central Documentation of genetic resources in animals in Germany* (TGRDEU) of the Federal Office for Agriculture and Food (13 pigs/litter, 2019) and earlier by Varona and colleagues^3^ (14.23 pigs/litter). The higher litter size observed here might result from differences in the genetic background of parent animals used and more generally from intensive breeding for increased ovulation rates and uterine capacity resulting in high litter size^37,38^. Negative effects of selection for larger litter sizes were stressed by various authors^5,14,39,40^, and include a general decrease in birth weight and an increased number of lightweight born piglets^7^. The mean birth weight observed in the present study showed a negative correlation (71%) to litter size. Compared to former data from our pig husbandry^41^, the mean birth weight and the weight of piglets categorized as low birth weight animals were lowered in piglets included in our study by 3.7% and 6.5%, respectively. Litters classified as large (>16 pigs/litter) showed an even more pronounced reduction (6.6% and 7.4%) compared to the study of Rehfeldt and colleagues^41^. In our study, in larger litters fewer piglets with heavy birth weight and significantly more low birth weight piglets were born. In larger litters, slightly more runt piglets were born, which are known to have a high neonatal morbidity and mortality^42^, and often have to be sacrificed. English and colleagues^43^ reported a postnatal piglet mortality of 40–50% during the first 2 days after birth, whereas runt piglets (<800 g birth weight) exhibited a mortality rate of 56.5%. Pomeroy^44^ even showed a mortality of 83% during the first 3 days after birth for piglets weighing less than 900 g at birth. LBW piglets are characterized by persisting growth impairment and altered body composition resulting in lower performance and suboptimal carcass quality^15,42,45^. In the litters assessed here, LBW piglets were not cross-fostered, like conducted in some studies^46–48^. Under these conditions, it became clear already at day 4 of life that NBW piglets were able to gain more body weight referred to their birth weight as LBW piglets.

Muscle growth, as the main component of body weight gain, depends on myofiber number and the increase in myofiber diameter^20,49^. In the pig, myofiber formation is mostly completed at birth^50^, and thus, postnatal muscle gain relates mainly to fiber hypertrophy^51^. Piglets classified as having LBW in this study exhibited previously described changes in muscle tissue characteristics like reduced concentrations of DNA and protein and reduced CK activity^15,52^. CK isoenzymes are upregulated during postnatal skeletal muscle development and lower CK activity also reflects a lower proportion of myofiber protein in LBW piglets^53^. A significantly reduced DNA/protein (P = 0.008) and RNA/protein level (P = 0.016) in SM muscle of LBW piglets compared to NBW piglets (data not shown) also points to a reduction in protein synthesis. For example, reduced levels of the structural proteins Keratin 10 and ß-Actin were found in the gastrocnemius muscle of newborn piglets with IUGR^54^. In conjunction with the reduced muscle fiber cross-sectional area observed in this study, our results reflect a retardation of postnatal hypertrophic growth in LBW compared to NBW piglets.

Growth retardation of LBW piglets might be related to energy deficiency. In our study, feed intake and time of suckling were not reported for the tested animals, but the observed elevation of triglyceride and NEFA concentrations in blood serum of LBW piglets point to a worse nutritional status of these animals^55,56^. LBW piglets have a competitive disadvantage in suckling over their heavier siblings^2,9,57^ and therefore, an insufficient intake of colostrum and transient milk, the main energy sources until day 4 of life^58–60^. On the other hand, body energy reserves are small in neonatal piglets and consist mainly of glycogen^58,61,62^. Total glycogen pools are more limited in LBW piglets and will be more rapidly depleted due to an increased requirement for energy^62,63^. As glycogenic substrates are required to fuel energy production from fat^58^, lower oxidation of fat from colostrum/milk is the most likely explanation to explain the higher NEFA levels found in LBW piglets. Newborn piglets have no fat reserves and a very low body fat content of about 2%^64^. In our study, the intramuscular fat content was below 0.05% and independent of birth weight, because it mainly presents structural fat in cell membranes which cannot be used for oxidation^58,64^. In neonatal piglets most available energy is needed for live keeping activities such as locomotion, suckling and thermoregulation^58,63^. In parallel, substantial energy is needed for fast muscle growth and maturation^65,66^.

To further characterize metabolic differences in LBW and NBW piglets, ICDH and LDH activities (measure for oxidative and glycolytic metabolism) were measured. As reflected by the low ICDH and high LDH activity, SM and LD muscles investigated here are light muscles in which energy is mainly produced via glycolysis^67^. Fast muscle fibers show low levels of oxidative enzymes and mainly use glycolysis for energy production^66,68,69^. Pig light muscles are mainly composed of glycolytic IIB myofibers (90%), and have a lower proportion of mixed IIA and oxidative type I fibers^67^. Thus, a main step in postnatal myofiber differentiation is the transformation of initially oxidative type II fibers to an anaerobic state of differentiation, which also leads to a drastic increase in fiber size^67,70^. Therefore, numerically lower LDH activity and smaller FCSA might reflect that the process of postnatal myofiber maturation is disturbed or slowed down in 4 day old LBW piglets.

Properties of muscle fibers not only depend on fiber type but show a high degree of metabolic plasticity^68,69^. ICDH enzymes catalyze the oxidative decarboxylation of isocitrate to α-ketoglutarate, during which NAD(P)H is produced^71^. ICDH concentration of SM and LD muscles is reduced in LBW piglets of our study. Due to negative effects on the development of cell membranes and on angiogenesis this could contribute to our findings of reduced growth, cell viability and muscle tissue capillarization^72^. In addition, lower ICDH concentration reflects a reduced oxidative capacity in muscles of LBW piglets. Wang and colleagues^54^ found a higher concentration of mitochondrial F1-ATPase in muscles of neonatal LBW piglets. F1-ATPase functions to hydrolyze ATP, thereby reducing its availability for ATP-depending pathways^73^. Under such conditions, reaction products of ATP hydrolysis and of the adenylate kinase reaction (ADP, AMP) are allosteric activators of key glycolytic enzymes and increase ATP production via glycolysis^74^. ADP and H^+^ generated by ATPase are also substrates of the muscular phosphocreatine (PCr)-creatine kinase (CK) system. Sarcomeric mitochondrial CK (MtCK) and cytosolic CK enzymes enable the synthesis of PCr from intramitochondrial ATP^75–77^. In addition, the inner mitochondrial membrane exchange of ADP and ATP is promoted by MtCK^76^. CK activity is significantly reduced in tissue of SM and LD muscles of LBW piglets. Thus, under conditions of high ATP turnover and limited oxygen availability, LBW piglets might have reduced abilities to maintain cytosolic ATP levels and to prevent H^+^ accumulation from net ATP hydrolysis^74,76,78,79^.

Energy and nutrient deficiency and associated metabolic changes can greatly affect the cellular components of muscle early postnatal development^80^. Of the various cell types (hematopoetic cells, microvascular cells, fibro/adipogenic cells, fibroblasts) found in muscle, SC are most critical^81–83^. SC are a source for myonuclei during myofiber growth and for life long maintenance and repair of muscle^32,70,84,85^. Thus, a main process of early postnatal muscle development is the formation of a SC reserve or stem cell pool^36^. However, the causal contribution of SC to the muscle phenotype observed in LBW piglets was only rarely analyzed and the heterogeneity within the common pool of SC was not considered^86^. In our study, two intrinsically distinct SC subpopulations were isolated from LBW piglets for the first time using a method described previously^36^. This approach opens up new possibilities because these populations have different functions in myogenesis and alterations in LBW piglets were possibly missed in mixed mass cultures investigated before.

From gene expression analyses it becomes clear that SC isolated from LBW piglets are generally able to undergo the myogenic program as shown by the expression of marker genes like *Pax7*, *Myf5*, *MyoD*, or *MyoG*. However, we found a significantly reduced MPC yield in muscles of LBW piglets affecting both cell subpopulations but the negative effect is stronger for the SPS population. SPS cells are thought to form the reserve cell compartment from which SPF cells can be supplied during postnatal growth and thus, are normally characterized by a lower ability to differentiate^36,87^. In our study, expression of *MyoG*, known to be essential for myoblast differentiation^88^, is increased in SPS cells from LBW piglets. In contrast, SPF cells of LBW piglets show a tendency to lower *MyoG* expression but a strong increase of *Myf5* expression compared with that from NBW piglets in our study. As SPF cells are proposed to be mainly responsible for fast hypertrophic growth of existing myofibers^36^, this could explain why myofiber growth of LBW piglets is reduced even though the number of myonuclei per fiber is similar in LBW and NBW piglets. Although increased *MyoG* expression in the SPS population can be seen as a compensatory response to ensure postnatal muscle growth, it also reflects premature differentiation of progenitor cells, thereby exhausting the muscle stem cell pool. In our previous study with NBW piglets^36^, expression of the myogenic determination gene *Myf5*^89^ has been shown to be upregulated in SPF compared with SPS cells. Here, *Myf5* expression seems to be further increased in SPF cells of LBW and higher than in NBW piglets. This points to the development of a SPF population more primed for myogenic commitment which could be important to retain their tissue identity^90^. However, taken into account the reduced myofiber growth observed in LBW piglets, it seems possible that despite expression of *Myf5* a higher proportion of SPF cells in LBW piglets does not enter the myogenic program. Indeed, Crist and colleagues^91^ found that SC can transcribe *Myf5* mRNA but can maintain quiescence by inhibition of *Myf5* translation via microRNA 31 thereby delaying the onset of myogenesis.

In addition, a Ca^2+^-dependent, Calcineurin- and NFAT-regulated upregulation of Myf5 has been observed specifically in quiescent myogenic cells of myotube cultures but not in myoblast cultures^92^. In our study, fewer Ki67 positive cells were found in muscle tissue of LBW piglets. Ki67 is expressed by cells which are in the G1/S/G2/mitotic phases of the cell cycle^93^. Thus, the lower proportion of Ki67+ cells in muscle tissue from LBW piglets can result from irreversible cell cycle exit due to differentiation and from a higher number of quiescent G0 cells being growth arrested. Indeed, viability was decreased in cells isolated from LBW piglets, although the same isolation protocol was used. Developing muscle structures have to become properly vascularized which is mainly regulated by VEGF (vascular endothelial growth factor)^94–96^. Here, we found a significantly lower capillary-to-fiber-ratio in LBW compared with NBW piglets probably resulting in inadequate blood supply, and thus, reduced delivery of oxygen, supply of nutrients and growth factors, and removal of metabolites^97^. It is known that the number of SC is directly associated to the number of capillaries^98,99^. In human and mouse the number of capillaries and SC associated with a myofiber correlates positively, as well^99^. Beyond this there is an extensive cross-talk between myofibers and adjacent blood vessels influencing each other bi-directionally in numerous ways. Spatial proximity seems to be a prerequisite since myonuclei and SC are mainly located close to blood vessels which was reported for several mammalian species, e.g., rat, dog, or human^96,100^. The decreased capillary-to-fiber-ratio in LBW piglets could further contribute to the reduced cell yield found after isolation of MPC. Whereas endothelial cells have been shown to stimulate SC proliferation, associated pericytes, which are embedded into the capillary basal lamina, promote the SC differentiation program and are important for angiogenesis, microvasculature structural integrity, and blood flow regulation^99,101–103^. Myogenic cells, on the other hand, were shown to act proangiogenic during differentiation^96^, possibly to ensure sufficient supplementation of the growing muscle.

In summary, combining new and updated data on energy metabolism, muscle capillarization and the abundance and characteristics of first-time isolated satellite cell subpopulations sheds new light on possible reasons for the lifelong growth retardation caused by low birth weight. A lower protein amount found in muscle tissue strongly contributes to a disturbed myofiber development. Although LBW piglets are in need for high demands of energy and nutrients, they have to cope with deficits as shown by an elevation of triglyceride and NEFA levels as well as a reduced capillary-to-fiber-ratio. Spending energy for life keeping actions seems to be prioritized above extensive muscle growth. Decreased capillarization might as well be associated with a reduced SC abundance found in LBW piglets. Gene expression analyses of SC subpopulations argue for a disturbance in postnatal myofiber maturation. Further studies on a cellular level, especially investigating possible differences in metabolic plasticity and myogenic differentiation potential between SC subpopulations, are needed to reveal underlying molecular mechanisms.

## Methods

### Animals

4-day-old female piglets of the German Landrace were used for the experiments. Animal husbandry in the experimental pig unit of the Leibniz Institute of Farm Animal Biology and slaughter followed the guidelines of the Animal Care Committee of the State Mecklenburg-Western Pomerania, Germany, based on the German Law of Animal Protection. Animals were either stunned by a captive bolt or anaesthetized by injection of Xylariem (0.3 ml/kg body weight, 20 mg/ml Xylazin, Ecuphar GmbH) and Ursotamin (0.4 ml/kg body weight, 100 mg/ml Ketamine, Serumwerk Bernburg) followed by exsanguination. As animals were not manipulated before slaughter, no animal experiment was conducted. Piglets for experimental analyses were selected following the criteria set in^52^, where a low birth weight was defined between 0.8–1.2 kg and normal birth weight between 1.20–1.62 kg. NBW and LBW piglets were selected from the same litter, respectively. Body weight at birth and at slaughter was monitored. To analyze litters of German Landrace pigs more general, data routinely collected in the experimental pig unit (e.g. (birth) weight and gender) were evaluated.

For all experiments (either using tissue, serum, or isolated cells), a total number of 50 NBW and 50 LBW piglets was necessary, which were sampled continuously within 46 months. All animals were kept under the same conditions and selected using the same criteria. It is not possible to perform all experiments presented here using the very same animals since obtained material would not be sufficient for all analyses in parallel. Siblings with normal or low birth weight are compared in every experiment to ensure continuity.

### Serum analysis

Slaughter blood samples were collected immediately after sacrifice and allowed to coagulate at room temperature. After centrifugation of liquid phase (10 min, 1,500 g, 4°C), serum was collected, frozen and stored until analysis. Serum levels of non-esterified fatty acids (NEFA), triglycerides and glucose were determined with the automatic enzymatic analyzer ABX Pentra 400 (Horiba Medical) as described before^55,104^ using commercial kits (NEFA-HR(2) Assay, WAKO Chemicals; Pentra Triglycerides CP, Axonlab AG; Pentra Glucose HK CP, Axonlab AG). The detection limit and inter-assay CV were as follows: NEFA 0.11 mmol/l, 0.9%; triglycerides 0.08 mmol/l, 1.4%, glucose 0.11 mmol/l, 0.9%.

### Analysis of chemical tissue composition

Composition of LD muscle tissue was analyzed after sacrifice. Dry matter and water content were determined by weighing the tissue before or after drying, respectively. Fat content was determined using ANKOMXT15 Extractor (ANKOM Technology); following an AOCS approved procedure (The American Oil Chemists’ Society, Am 5–04).

### Histological analyses

To further characterize the developmental status of piglets with LBW in comparison to their siblings with NBW, muscle tissue samples were taken at day 4 of life immediately after slaughter. Information on muscle fiber development, capillarization, proportion of connective and fat tissue, as well as the number of proliferating muscle cells was determined.

Muscle samples were dissected from the middle of the right *musculus semimembranosus* (SM) or *musculus longissimus dorsi* (LD) and immediately frozen in liquid nitrogen. Serial transverse 10 µm sections were cut at −20 °C using a cryotome (Leica Biosystems) and stained with eosin to count the total fiber number and to quantify muscle and connective tissue area. Intramuscular adipose tissue area was quantified after Oil Red staining. Alkaline phosphatase (ALP) was visualized to count capillaries within muscle tissue. To assess proliferation, cryosections were stained with a rabbit anti-Ki67 antibody (undiluted, Biologo, KI505) and subsequently with a rabbit anti-Laminin antibody (1:500, Agilent Technologies, Z0097). A biotinylated horse anti-rabbit antibody and the Vectastain ABC HRP Kit (Vector Labs) were used. Cell nuclei were stained with methyl green (Zytomed Systems, ZUC002) before samples were mounted using the Roti-Histokitt (Carl Roth). For all quantifications ten randomly selected fields per sample were analyzed. Only Ki67+ cell nuclei of which at least half of the nucleus was found within a muscle fiber, where counted as positive.

### Biochemical analyses of muscle tissue

Muscle samples for biochemical analyses were dissected from the middle of the right SM and LD muscle and immediately frozen in liquid nitrogen. 100 mg muscle were pestled in liquid nitrogen and homogenized mechanically in 0.01 M potassium phosphate buffer containing 1 mM EDTA. Homogenates were centrifuged (14,000 g, 15 min, 4 °C) and the supernatant was used for further analysis.

DNA was labeled with Hoechst 33528 (Sigma-Aldrich) and the amount was determined fluorometrically by a fluorescence reader (FLx800, Bio-Tek Instruments) against a calf thymus DNA standard (Sigma-Aldrich)^105^. RNA was quantified fluorometrically using SYBR Green II RNA Gel Stain (MoBiTec) against a calf liver RNA standard (Sigma-Aldrich). Total protein amount was measured according to the method described by Peterson^106^.

Creatine kinase (CK) activity was assessed using the CK-NAC-Hit kit (IFCC method, BIOMED Labordiagnostik GmbH) according to the manufacturers’ instructions. For lactate dehydrogenase (LDH) supernatant was incubated with 0.19 mM NADH, then 0.757 mM Na-pyruvate was added to measure NADPH photometrically and to calculate LDH activity using a NADPH standard curve. For determination of isocitrate dehydrogenase (ICDH) activity 4 mM isocitrate, 3.3 mM MnSO_4_ and 0.35 mM NADP were added to each sample to measure NADPH photometrically. Enzyme activity was calculated against a NADPH standard curve. All amounts/activities are given per g muscle.

### Isolation and cultivation of myogenic cells

The isolation of myogenic cells was performed as described earlier^36^. Briefly, SM and LD muscle were dissected, trimmed, digested with trypsin 1x solution (4000 U/ml, Sigma-Aldrich), and filtered. The amount of trypsin was adjusted to the amount of muscle. Via gradient centrifugation using layers of 70%, 50%, 40%, and 25% Percoll, cells were enriched and separated into two subpopulations. *Subpopulation fast* (SPF) was collected at the 40%/50%-interface and *subpopulation slow* (SPS) was collected at the 50%/70%-interface. Cells were resuspended in growth medium (αMEM Eagle containing 20% FBS, 100 U/ml penicillin/streptomycin, 2.5 µg/ml amphotericin, 0.05 mg/ml gentamycin, all purchased from PAN Biotech). Cell number and viability were determined using the Countess Automated Cell Counter (Thermo Fisher Scientific) via trypan blue staining.

### Gene expression analysis

Gene expression was analyzed as described before and previously obtained data for NBW piglets are partly included in this study^36^. Briefly, freshly isolated cells were resuspended in RNAprotect Cell Reagent (Qiagen). Total RNA was isolated using the NucleoSpin RNA kit (Machery-Nagel) according to the manufacturer’s instructions. The quantity of RNA was assessed at 260 nm with a Nano-Photometer (Implen). RNA quality was controlled by using RNA 6000 Nano kit and the Bioanalyzer 2100 (both from Agilent). RNA samples with a RIN of at least 8 were reverse transcribed to cDNA (500 ng/20 µl reaction) with iScript cDNA Synthesis kit (Bio-Rad). Quantitative real-time PCR (qRT-PCR) was carried out in an iCycler iQ Real-Time PCR Detection System (Bio-Rad). Samples were run in triplicates. The final volume of the reaction (20 µl) was composed of 10 µl iQ SYBR Green Supermix (Bio-Rad), the gene specific primers (1 µl of 20 pmol/µl each) and 10 ng of DNA. The temperature program consisted of an initial denaturation at 95 °C for 3 min followed by 40 cycles of 95 °C for 15 s, 60 °C for 1 min, and 60 °C for 30 s. PCR was followed by a melting curve analysis (10 min, 55 °C–94.5 °C) to validate specificity. Primers were previously published: *scPax7*, *scMyf5*, and *scMyoD1*^107^, *scDesmin*^108^; *scMyoG and MyH2*^109^; *eMyH*, *MyH1*, and *MyH4*^110^. The C_q_ values of the target genes were normalized to the references genes *Top2b* and *Gapdh*^111^, and *Yhwaz*^36^. Relative gene expression was calculated by using the 2^−ΔΔCq^ method^112^. Negative controls without cDNA template were included in all reactions.

### Statistical methods

For general litter analyses (see Table 1) piglets were assigned into three birth weight groups according to quartiles of frequency distribution (IBM SPSS Statistics 22): 25% low birth weight (0.8–1.15 kg), 50% middle birth weight (1.16–1.54 kg) and 25% high birth weight (>1.54 kg). Runts with a birth weight <0.8 kg were excluded from this categorization. In sum 1058 piglets were analyzed; piglets born alive as well as dead born piglets were included.

SigmaPlot 13.0 (Systat Software Inc.) was used for all other statistical analysis. When normality test (Shapiro-Wilk) and equal variance test (Brown-Forsythe) were passed, a (paired) t-test was performed. If one of the tests was not passed, Mann-Whitney Rank Sum test, Wilcoxon Signed Rank Test (for comparing two conditions) or Kruskal-Wallis one way ANOVA on Ranks (Student-Newman-Keuls Method) was performed. All graphs show mean ± SD if not stated otherwise in the figure legend. Statistical significances were assigned as follows: ***P ≤ 0.001, **/##P ≤ 0.001, *P ≤ 0.05, trend (T) P ≤ 0.1.

## Data Availability

The datasets generated during and/or analyzed during the current study are available from the corresponding author on reasonable request.
